# Cognitive impact of neuronal antibodies: encephalitis and beyond

**DOI:** 10.1038/s41398-020-00989-x

**Published:** 2020-09-01

**Authors:** L. L. Gibson, A. McKeever, E. Coutinho, C. Finke, T. A. Pollak

**Affiliations:** 1 Department of Old Age Psychiatry, Institute of Psychiatry, Psychology and Neuroscience, King’s College London, London, UK; 2 University of Cambridge, Cambridge, UK; 3 Department of Basic and Clinical Neuroscience, Institute of Psychiatry, Psychology and Neuroscience, King’s College London, London, UK; 4 MRC Centre for Neurodevelopmental Disorders, King’s College London, London, UK; 5 Department of Neurology, Charité – Universitätsmedizin Berlin, Berlin, Germany; 6 Berlin School of Mind and Brain, Humboldt-Universität zu Berlin, Berlin, Germany; 7 Department of Psychosis Studies, Institute of Psychiatry, Psychology and Neuroscience, King’s College London, London, UK

**Keywords:** Prognostic markers, Diseases

## Abstract

Cognitive dysfunction is a common feature of autoimmune encephalitis. Pathogenic neuronal surface antibodies are thought to mediate distinct profiles of cognitive impairment in both the acute and chronic phases of encephalitis. In this review, we describe the cognitive impairment associated with each antibody-mediated syndrome and, using evidence from imaging and animal studies, examine how the nature of the impairment relates to the underlying neuroimmunological and receptor-based mechanisms. Neuronal surface antibodies, particularly serum NMDA receptor antibodies, are also found outside of encephalitis although the clinical significance of this has yet to be fully determined. We discuss evidence highlighting their prevalence, and association with cognitive outcomes, in a number of common disorders including cancer and schizophrenia. We consider mechanisms, including blood-brain barrier dysfunction, which could determine the impact of these antibodies outside encephalitis and account for much of the clinical heterogeneity observed.

## Introduction

An expanding array of pathogenic neuronal autoantibodies are being identified, each targeting different neuronal surface antigens and thought to cause distinct and clinically recognisable encephalitic syndromes. These antigenic targets have wide-ranging properties and distributions in the central nervous system but common to almost all autoimmune encephalitides is cognitive dysfunction. The nature of cognitive impairment and associated neuroimaging findings varies between syndromes and gives insight into the antibody-mediated mechanism of action. Neuronal autoantibodies have also been reported—so far mainly in the peripheral blood—in individuals without frank encephalitis. Although their significance outside the encephalitic context is not yet clear, there is a growing body of evidence to suggest these autoantibodies have pathogenic potential even in the absence of the encephalitic syndrome. In this review, we outline the cognitive profile of each of the commonest autoantibody-mediated encephalitides and consider the role of neuronal antibodies outside encephalitis. While a treatment of the neurotransmitter basis of cognition is beyond the scope of this review, it is important to note that these autoantibodies largely serve to disrupt the signalling transmission of neurotransmitters such as glutamate and γ-aminobutyric acid (GABA) which are integral to cognition. Glutamate is a ubiquitously distributed excitatory neurotransmitter that also acts as an intermediary in cerebral metabolism; the ionotropic glutamate-specific N-Methyl-d-aspartate receptor (NMDAR) and α-amino-3-hydroxy-5-methyl-4-isoxazolepropionic acid (AMPA) receptors are vital components of long-term potentiation (LTP) and long-term depression, processes understood to be the major synaptic substrates of learning and memory; changes in the neuronal surface density of these receptors therefore have direct effects on neuronal signalling with downstream impact on brain connectivity and cognitive processes. GABA receptors are present as ionotropic (GABA_A_ receptor) and metabotropic (GABA_B_ receptor) postsynaptic receptors that are bound by GABA, the major inhibitory neurotransmitter in the central nervous system (CNS). While long-range GABAergic neurons do exist, the majority of research attention relevant to cognition has focused on GABAergic interneurons, which appear to have a central role in the synchronisation of network activity and the generation of oscillations in different frequency bands, processes felt to facilitate the efficacy of information processing^1^.

Furthermore, the inhibitory-excitatory balance that is emergent from dynamic interactions of glutamatergic and GABAergic signalling is thought to play an important role in stimulus representation and information propagation and therefore is likely to be crucial not only for cognition but for behavioural processes defined more broadly^2,3^.

## Autoimmune encephalitis

Detailed neuropsychological characterisation is often challenging in the acute phase of autoimmune encephalitis due to the severity of clinical symptoms. Accordingly, in the acute phase clinical descriptions tend to be qualitative and the more extensive cognitive testing possible in the post-acute and chronic phases is frequently authored from a neurorehabilitation perspective, potentially introducing a selection bias towards cases with more severe dysfunction. While we describe the acute and chronic cognitive deficits separately, in practice such distinctions are not so easily delineated and there is often significant overlap. Table 1 summarises the cognitive impairment associated with each neuronal autoantibody-associated encephalitis. It is useful to note at the outset that while most autoimmune encephalitides are named after the putatively pathogenic antibody, there is increasing evidence that there may be variability in the breadth of the antibody response between these disorders; for example, in LGI1 encephalitis the polyclonal antibody response appears only to target the LGI1 protein^4^, whereas in NMDAR encephalitis less than 10% of intrathecal antibody-secreting cells are specific for the NR1 subunit of the NMDAR^5^. This raises the possibility that antibodies targeting other (non-canonical) epitopes, or even entirely different proteins, may contribute to the clinical expression of disease in some disorders.

**Table 1:** Cognitive impairment as a feature of neuronal autoantibody-associated encephalopathy in the acute phase and at long-term follow-up.

Antigen	Antigen description	Acute phase			Post-acute phase	
		Characteristic features of syndrome	Cognitive impairment	Specific domains of memory impairment	Cognitive impairment	Details of memory impairment
NMDAR (NR1 subunit)	Ligand-gated ion channel subunit	Frequent influenza-like prodrome followed by psychosis, anxiety, cognitive impairment, catatonia, seizures, movement disorders, autonomic dysfunction and reduced consciousness^6^	Deficits in across all domains; memory, information processing, attention, executive function, language, visuospatial processing and social cognition all affected^7,8^	Episodic and working memory impairment^14^. Delayed verbal memory, short term memory and visual memory particularly affected^13^	Episodic memory, processing speed and executive function remain impaired^7,8,13,14^	Greatest deficit in episodic and delayed verbal memory^13,14^
LGI1	VGKC- and AMPAR-associated secreted molecule	Features of limbic encephalitis; amnesia and seizures are the most common hallmarks. Psychiatric symptoms, sleep disturbances and hyponatraemia are also seen^34,40^. FBDS are specific for LGI1 encephalitis and frequently occur before cognitive dysfunction^33,42^	Disorientation and global confusion is typical with autobiographical memory impairment^34^	Particular impairment to autobiographical memory^34^	Few return to baseline cognition^40,42^. Prominent memory deficits remain with spatial disorientation^42^. Executive function, attention, semantic and phonemic fluency is also impaired^40,55^	Verbal, visuospatial and working memory deficits persist^40,55^
CASPR2	VGKC-associated adhesion molecule	Associated with a more diverse clinical presentation. Similar features of limbic encephalitis are often seen; seizures, cognitive impairment, personality change^36^. Neuromyotonia and autonomic dysfunction more common in CASPR2 encephalitis^34^. Also associated with Morvan’s syndrome where low titre LGI1 antibodies are also often present^30^.	Cognitive dysfunction is common with memory impairments but confusion and behavioural disorders are less prominent^36^	Anterograde and episodic memory disorders are typically seen^36^	Long term cognitive outcomes for CASPR2 encephalitis are not clear. For VGKC encephalitis (with LGI1 vs CASPR 2 not specified): a persistent memory deficit is seen, but executive function and processing speed recover following immunotherapy^39^.	Not clearly defined for CASPR2 encephalitis but in VGKC encephalitis (LGI1 and CASPR2 antibodies not distinguished) verbal memory is impaired^39^.
AMPAR	Ligand-gated ion channel	Diverse presentation including symptoms of limbic encephalitis. May present with prominent memory impairment, confusion, seizures or fulminant encephalitis^70^.	Impaired memory is the most common deficit, often with confusion and executive functioning impairments^70^. May also present with isolated amnesia^70,71^.	Anterograde memory loss^70^	Improves with immunotherapy (and tumour control when paraneoplastic). Memory deficits persist in some, worst outcomes in those presenting with fulminant encephalitis^70^.	Not reported
GABA_A_R	Ligand-gated ion channel	Diverse presentation including features of limbic encephalitis. Seizures almost always present and cognitive/behavioural symptoms are seen in two-thirds of patients^81^.	Memory deficits and confusion are less consistently reported in GABA_A_R encephalitis (27%)^82^.	Not reported	No published neuropsychological long-term outcomes	
GABA_B_R	G-protein coupled receptors	Diverse presentation including features of limbic encephalitis. Seizures, cognitive and behavioural symptoms almost univerally seen^90^.	Memory deficits and confusion present in many with GABA_B_R antibodies (47%)^88^. GABA_B_R associated with small cell lung carcinoma and may present with rapidly progressive dementia^90^.	Not reported	No published neuropsychological long-term outcomes	

### NMDAR encephalitis

#### Acute phase

NMDAR encephalitis is both the most common and best-defined cause of autoimmune encephalitis. Its onset is often heralded by an influenza-like prodrome followed by a characteristic progression from psychotic symptoms and cognitive impairment to seizures, movement disorder, autonomic instability and loss of consciousness^6^. While most frequently described in women of child-bearing age, it is increasingly recognised in children and older adults of both sexes^6^.

Cognitive dysfunction is often profound and in the acute phase typically extends across all domains. Deficits in executive function and memory are most marked but attention, language, visuospatial processing and social cognition are also affected to varying degrees^7,8^. Atypical, unusual presentations of cognitive impairment are occasionally described in NMDAR encephalitis; case reports illustrate disruption to temporal orientation with a loss of age awareness and also transient epileptic amnesia, characterised by repeated, brief episodes of anterograde and retrograde amnesia^9,10^. Another recent case report documented a patient who, due to the nature of their presentation with memory loss, cognitive fluctuations, visual hallucinations and sleep disorder, was initially misdiagnosed with Lewy Body Dementia before NMDAR antibodies were identified leading to effective immunotherapy treatment^11^. In older adults with NMDAR encephalitis cognitive impairment is often more prominent which may account for some of the variability seen^12^.

#### Long term follow-up

While cognitive dysfunction is extensive in the acute phase it can also persist for years after the initial insult^13^. As in the acute phase, episodic memory and executive function are most consistently affected 1 year after initial presentation^7,8,13,14^. A recent systematic review found chronic cognitive impairment in up to three quarters of patients, with timely immunotherapy the most important factor determining positive outcomes^8^. This emphasises the persistent and major morbidity of cognitive impairment in anti-NMDAR encephalitis for patients, in addition to the importance of early diagnosis and appropriate treatment^13^.

#### Mechanisms underlying cognitive impairment

The NMDA receptor is a tetrameric ligand-gated ion channel which mediates excitatory transmission in the CNS and is crucial for LTP, the neural substrate for learning and memory. In NMDAR encephalitis, there is substantial intrathecal production of IgG NMDAR antibodies which target the NR1 subunit of the receptor, causing a reversible and titre-dependent internalisation of the NMDA receptor with subsequent reduced receptor density and reduction in NMDAR-mediated currents^15,16^. The antibody-mediated disruption of the interaction between the NMDA and ephrin-B2 receptor is central to this, causing the NMDAR to become displaced which allows subsequent internalisation^17^. Indeed, animal studies suggest that if ephrin-B2 is co-administered, the pathogenic effects of the antibodies are blocked and no downstream effects are seen^18^.

Accordingly, NMDAR-dependent LTP is depressed in mouse hippocampal slices with prolonged exposure to CSF from patients with NMDAR encephalitis^19^. Furthermore, in vivo infusion of NMDAR antibodies reduces excitatory postsynaptic currents in rat hippocampal neurons and simultaneously impairs learning and spatial working memory^20^. Infusion of inflammatory cytokines also reduced excitatory postsynaptic currents and led to further impairment in learning performance suggesting there may be other additive factors influencing cognitive dysfunction^15^. More recently, object recognition has been shown to be impaired following injection of NMDAR antibodies to rat hippocampi, widening the application of NMDAR antibody-mediated cognitive dysfunction^21^. NMDAR antibodies cause a dose-dependent increase in extracellular glutamate, akin to the elevated level of glutamate following ketamine administration which is associated with cognitive effects^22^.

**Fig. 1:**
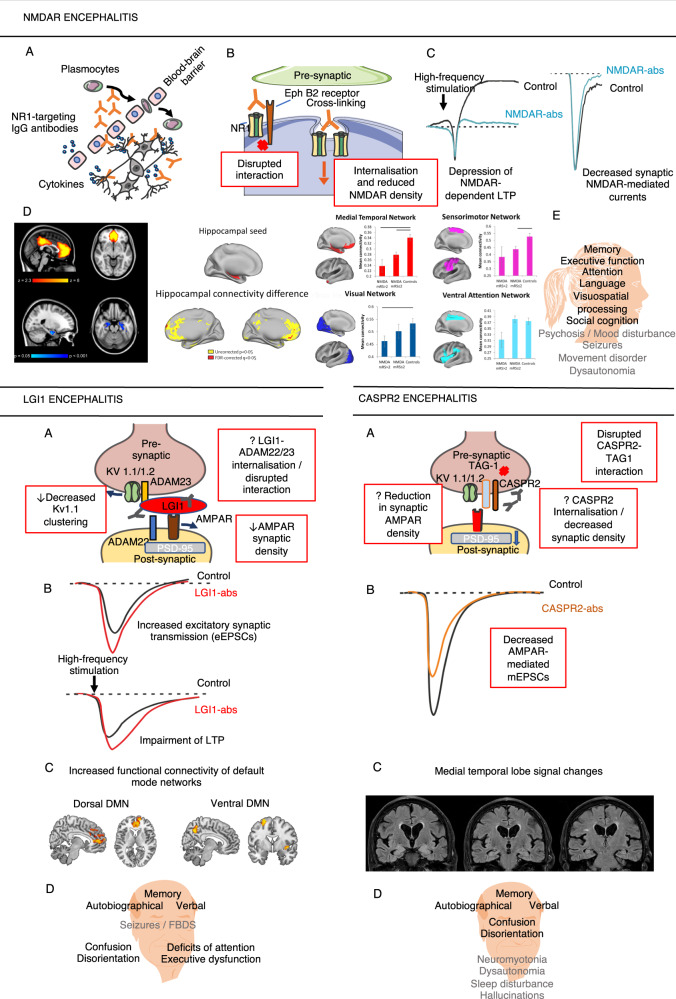
NMDAR encephalitis. **a** NMDAR-antibody access to the brain; **b** Molecular mechanisms of pathogenicity; **c** Functional effects of the NMDAR-antibodies; **d** Functional connectivity changes in NMDAR encephalitis. (Left) Impaired functional connectivity between the hippocampus and the medial prefrontal cortex identified using independent component analysis and dual regression. The severity of hippocampal functional connectivity impairment correlates with individual memory deficits. (Middle) Impaired connectivity of the hippocampus with the medial prefrontal cortex replicated using a seed-based approach. In addition, reduced hippocampal connectivity with other regions of the default mode network was observed, e.g. the posterior cingulate cortex and the precuneus. (Right) Using a network-based approach, significantly reduced functional connectivity was found within several large-scale networks, including the medial temporal lobe network, the sensorimotor network and the visual network. [Figures reproduced with permission from^24,25^] **e** Clinical phenotype. **LGI1 encephalitis:** **a** Molecular mechanisms of pathogenicity; **b** Functional effects of the LGI1-antibodies; **c** Patients with LGI1 encephalitis had increased functional connectivity of the dorsal and ventral default mode network (DMN) that correlated with working memory (dorsal DMN) and episodic memory (ventral DMN) performance. [Figures reproduced with permission from^55^]; **d** Clinical phenotype. **CASPR2 encephalitis and Morvan’s Syndrome:** **a** Molecular mechanisms of pathogenicity; **b** Functional effects of the CASPR2-antibodies; **c** Bilateral FLAIR hyperintense signal and mild atrophy of the hippocampus in a patient with CASPR2 encephalitis; **d** Clinical phenotype. Components of this figure were created using Servier Medical Art templates, which are licensed under a Creative Commons Attribution 3.0 Unported License; https://smart.servier.com. The shapes of the electrophysiological traces were modelled on data published in^47,61,147^.

NMDARs are highly concentrated in the hippocampus and frontal cortex which likely underlies the predominance of cognitive deficits in episodic memory and executive function^23^. Indeed, reduced functional connectivity between the hippocampus and the medial prefrontal cortex and impaired connectivity within the medial temporal lobe (MTL) network was observed in patients with NMDAR encephalitis and shown to predict the severity of memory impairment (Fig. 1)^24,25^. Furthermore, although the disorder is not a classical limbic encephalitis, in the post-acute phase reduced bilateral hippocampal volume and microstructural integrity is observed, which likewise correlates with memory impairment^26^. Widespread damage to superficial white matter – which encompasses short-range association fibres and intracortical myelin—additionally contributes to impairments of attention and memory^27^, and extensive changes in deep white matter integrity correlate with disease severity^24^. This structural damage to hippocampus and white matter suggests pathological mechanisms ongoing beyond the demonstrated reversible internalisation of NMDAR without damage to neurons;^16,26^ T cell mediated processes may have an as-yet under-appreciated role here^28^.

### Encephalitis formerly attributed to antibodies to the voltage gated potassium channel (VGKC): LGI1 and CASPR2 encephalitis

The first potentially reversible, immunosuppression-responsive form of limbic encephalitis was described nearly 20 years ago^29^. The VGKC antibodies originally detected were believed to directly target the Kv1.1, 1.2 and 1.6 channels. However, it is now understood that pathological VGKC antibodies target the extracellular domains of one or more of three proteins tightly complexed with VGKCs; LGI1, CASPR2 and contactin-2, each with different implications^30^. “Double negative” VGKC antibodies—that is VGKC antibody positivity without LGI1 or CASPR2 positivity—are of questionable clinical significance (potentially targeting intracellular targets) and for this reason VGKC antibodies should not be routinely tested in the initial investigation of a patient with a suspected autoimmune CNS disorder^31,32^.

### LGI1 encephalitis

#### Acute phase

Typical presenting symptoms of LGI1 encephalitis are those of a limbic encephalitis; symptoms include cognitive impairment, behavioural changes and focal seizures. Seizures typically precede the onset of cognitive impairment, with a progressive amnesia usually developing at their crescendo^33^. Unlike other autoimmune encephalitides (and most autoimmune conditions) LGI1 encephalitis is most common in middle-aged males. Although LGI1 encephalitis is phenotypically similar to other paraneoplastic and non-paraneoplastic limbic encephalitides, faciobrachial dystonic seizures (FBDS) are unique to LGI1 encephalitis and are a useful clinical differentiator^34^.

Prominent amnesia is a hallmark of limbic encephalitis associated with LGI1-antibodies; autobiographical memory is particularly impaired, often with significant confusion and disorientation^30,35,36^. Isolated amnestic syndrome can occur in up to 10% of cases of LGI1 encephalitis^35^ and, in the absence of seizures and where the onset is insidious, LGI1 encephalitis can mimic other syndromes of cognitive impairment such as neurodegenerative dementias^37^. Indeed, numerous case reports have documented LGI1 encephalitis misdiagnosed as Alzheimer’s disease, Creutzfeld-Jacob Disease and Dementia with Lewy Bodies before further investigation elucidated the true cause and led to reversal of the cognitive impairment with immunotherapy^38^.

#### Long term follow-up

Looking at long term outcomes for patients with previously defined VGKC antibodies collectively, without differentiating LGI1 or CASPR2-positive patients, cognitive deficits correlate with antibody titre and are most marked for verbal memory, while processing speed and executive function are relatively spared^39^. In LGI1 encephalitis, most patients have a chronic cognitive impairment, with memory predominantly affected but deficits of attention and executive function also reported^40–42^. Greater disease severity, delays to immunotherapy or longer courses of immunotherapy (likely mandated by greater disease severity) are all associated with more profound cognitive dysfunction in LGI1 encephalitis^40^. Indeed, early treatment of isolated FBDS significantly reduces the risk of developing cognitive impairment, highlighting the importance of recognising FBDS early^43,44^.

#### Mechanisms underlying cognitive impairment

Unlike NMDAR antibodies, LGI1 antibodies are predominantly of the IgG4 subclass, although the IgG1 subclass may also contribute to pathology. LGI1 is a trans-synaptic protein which complexes presynaptic ADAM23 with postsynaptic ADAM22. LGI1 antibodies act directly to disrupt this binding which subsequently reduces synaptic AMPAR density^45^. However, given the marked differences in clinical presentation with AMPAR encephalitis, it is likely that LGI1 antibodies have other downstream effects in addition to modulation of AMPARs. In one study of 103 patients, IgG1 antibodies occurred more frequently in patients with cognitive impairment, suggesting a role for complement-mediated pathology in this symptom domain^43^. Serum IgG VGKC antibodies from patients with limbic encephalitis led to cell excitability with an increased tonic rate of firing and strengthened mossy fibre-evoked synaptic responses in CA3 rat hippocampal slices—the sera used in this study was later found to have LGI1 rather than CASPR2 antibodies^46^. A selective VGKC antagonist mimicked these effects suggesting that the antibody-mediated increase in cell excitability is directly related to reduction in VGKC function. Petit-Pedrol et al. later demonstrated LGI1 antibody-mediated reductions in synaptic density of both K_v_1.1 VGKC and AMPAR receptors with simultaneous, reversible memory deficits^47^. They observed hyperexcitability, increased glutamatergic transmission and reduced synaptic failure rate with a severe impairment to neuronal plasticity and LTP in CA1 and the dentate gyrus. A recent study further explored the pathogenic mechanisms of LGI1-antibodies and suggested the possibility of synergistic contributions. Monoclonal antibodies derived from patients with LGI1-mediated disease were found to target multiple epitopes, with distinct specificities to the protein’s LRR and EPTP-domains. In this study, LRR and EPTP-binding LGI1 antibodies seemed to mediate distinct functional effects. LRR-directed antibodies bound and internalised the LGI1-ADAM22/23 complexes. Surprisingly, this mechanism was independent of IgG bivalency, as both IgG4 antibodies and Fab fragments retained this capacity. The dominant effect of EPTP-binding antibodies, on the other hand, appeared to be disruption of the interaction between LGI1 and its receptors. Both LRR- and EPTP-specific antibodies completely abrogated LTP induction at CA3-CA1 hippocampal synapses, which translated to impairment of recognition memory in mice exposed to LRRP-antibodies^48^. These findings likely explain the amnestic syndrome experienced by patients with LGI1 encephalitis.

Interestingly, in humans, LGI1 gene mutations lead to autosomal dominant lateral temporal lobe epilepsy characterised by frequent partial seizures often with auditory auras (rather than FBDS) but typically without cognitive impairment^49^. This might relate to differences in the timing of protein dysfunction (from early neurodevelopment vs later in adult life) or to other in vivo roles of LGI1 and its targeting by autoantibodies that have not yet been clearly defined.

In the rodent, LGI1 and CASPR2 are preferentially expressed in CA3 and CA1 hippocampal subfields^30,50^. Correspondingly, MR imaging in the acute phase of LGI1 or CASPR2 encephalitis is usually abnormal, typically showing inflammation in MTL, an area critically involved in memory processing^51,52^. At follow-up, almost all patients show some degree of hippocampal atrophy, which is often bilateral, with loss seen particularly in the CA3 subfields^40,53^. The volumetric atrophy also correlates with the severity of episodic autobiographical and verbal memory impairments^40,53^. The microstructural integrity of the hippocampus is also impaired on a wider level which associates with both disease severity and memory function; functional connectivity of the remaining hippocampus correlates closely with the degree of memory impairment^40,54^. Interestingly, functional connectivity analyses also show characteristic alterations in several large-scale networks, suggesting that LGI1 encephalitis is not confined to the limbic system. Increased connectivity in the ventral and dorsal default-mode network is associated with improved memory performance, indicating a compensatory mechanism, while connectivity in the salience network is reduced and correlated with impaired memory function^55^. These network changes indicate cognitive deficits beyond mere memory impairment in LGI1 encephalitis, suggesting brain-wide alteration of the connectome triggered by focal hippocampal damage.

### CASPR2 encephalitis and Morvan’s syndrome

#### Acute phase

CASPR2 antibodies associate with a wide range of neurological syndromes, which often overlap in the same patient. Manifestations include peripheral nerve hyperexcitability (often referred as neuromyotonia), neuropathic pain, paroxysmal movement disorders and limbic encephalitis^56^. Among the different CASPR2-related syndromes, limbic encephalitis and Morvan’s syndrome have a particular impact on cognitive functions. CASPR2 encephalitis, similarly to LGI1 encephalitis^57^, is characterised by limbic dysfunction, with temporal seizures, memory impairment and frontal dysfunction^36^. Morvan’s syndrome, a rarer disorder, is characterised by peripheral nerve hyperexcitability and encephalopathy, in addition to sleep disturbance, hallucinations, dysautonomia and pain. Whether Morvan’s syndrome is a distinct entity or merely a combination of autoimmune encephalitis and peripheral nerve hyperexcitability is unclear, but some suggest there are sufficient differences to consider it a distinct syndrome. Limbic dysfunction, such as temporal seizures, anterograde amnesia or hyperintensities in the MRI are uncommon in Morvan’s syndrome, except in patients who are both CASPR2 and LGI1-antibody positive. Both CASPR2 limbic encephalitis and Morvan’s syndrome are more common in elderly male patients, but association with thymoma and other autoimmune diseases is much more common in Morvan’s syndrome.

#### Long term follow up

Data on long term outcomes, particularly on cognitive sequelae, are remarkably scarce in CASPR2 encephalitis and Morvan’s syndrome, and it is often pooled together with that of LGI1 encephalitis or under the previous umbrella designation “VGKC encephalitis”. Partial or full recovery after immunosuppression is usually the norm in non-paraneoplastic CASPR2 encephalitis or Morvan’s syndrome^36,57^. However, relapses are frequent, particularly in the form of increased seizure activity, and are usually steroid-responsive.

#### Mechanisms underlying cognitive impairment

CASPR2, a member of the neurexin family, is a cell adhesion transmembrane protein first identified in the VGKC clusters (mainly Kv1.1 and Kv1.2) at the juxtaparanodes of myelinated neurons^59^. CASPR2 stabilises the VGKCs such that antibody-mediated disruption of this protein causes peripheral hyperexcitability syndromes. The role of CASPR2 at the CNS synapse, however, is not well known, but the bulk of data suggests important functions in synaptic processes and neuronal activity. CASPR2 was implicated in the trafficking of AMPA receptors to the synaptic membrane^60,61^, suggesting that glutamatergic transmission dysfunction could underpin the cognitive impairment seen in CASPR2-mediated CNS disease. Others suggest that CASPR2 has a role in inhibitory hippocampal synapses, and that the antibody-mediated perturbation of inhibitory interneuron activity could lead to increased neuronal hyperexcitability and ultimately to the seizures suffered by these patients^62,63^.

Like LGI1-antibodies, CASPR2-antibodies are predominantly of the IgG4 subclass, although IgG1 antibodies can also be present and are potential contributors to pathology. They too target multiple epitopes, with the protein’s N-terminal discoidin-like and laminin G1 domains being obligatory epitopes^36,64^. The mechanisms by which these antibodies cause disease, however, are still not completely understood and there have been conflicting results in the literature. While some report absence of CASPR2 internalisation^63,65^, others suggest that CASPR2 is indeed internalised^35,61,66^ by the antibodies. It is possible that the IgG subclass (IgG1 vs IgG4) titre differences in the CASPR2-IgG preparations used in these studies, as well as differences in the in vitro systems used, partially account for these contradicting results. Those groups that found no evidence for antibody-mediated internalisation suggest that CASPR2-antibodies exert their function through interference with the interaction between CASPR2 and TAG-1^63,65^. Finally, CASPR2-antibodies may also exert their pathogenicity by altering the protein’s known function in AMPAR synaptic traffic. A recent study showed significant synaptic loss of AMPARs in cortical neurons incubated with IgG1 and IgG4 CASPR2 antibodies, while in vivo injection of the same antibodies in the mouse visual cortex significantly decreased AMPAR-mediated currents^67^.

These results suggest that CASPR2 likely has different functions in different synapses, which would imply different but synergistic effects of the CASPR2-antibodies in the pathophysiology of encephalitis or Morvan’s syndrome. More studies on the pathogenic mechanisms of CASPR2-antibodies are necessary.

## Other neuronal autoantibody-mediated encephalitis

### AMPAR encephalitis

#### Acute phase

Due to its rarity, the clinical course of AMPAR encephalitis is not yet well characterised. Most studies describe limbic dysfunction at onset characterised by anterograde and retrograde amnesia, confusion, psychiatric symptoms and seizures^68^. First described in a series of ten patients, it was reported to mostly affect older women, often with an underlying malignancy and high rates of relapse^69^. The phenotype has since widened with marked heterogeneity in presentation observed^68,70^. However, cognitive dysfunction remains a universally prominent feature and isolated amnesic syndromes have also been observed with a focal impairment to anterograde memory^70,71^. Indeed, in a review of 18 cases there was evidence of cognitive impairment in all, ranging from anterograde memory impairments and executive dysfunction to generalised confusion^68^. A recent systematic review identified 55 patients with AMPAR encephalitis; a diverse phenotype was observed but amnesia was recognised as the most common clinical symptom^71^. However, amnesia at onset was also associated with greater diagnostic delays, highlighting the need for improved recognition of the symptomatic profile associated with AMPAR encephalitis.

#### Long term follow-up

There are limited data available on the neuropsychological outcomes of AMPAR encephalitis patients. Case reports have described considerable neurocognitive improvement at follow-up with a third achieving complete recovery, but this has yet to be characterised quantitatively^72^. Although in general outcomes appear favourable, psychiatric symptoms or fulminant encephalopathy at onset is associated with poor prognosis at follow up^70,72^. In a case report, significant memory impairment was found to persist 1 year after disease onset, accompanied by hippocampal atrophy, persistent hippocampal hypermetabolism in ^18^FDG PET imaging and ongoing epileptic activity on EEG^73^.

### Mechanisms underlying cognitive impairment

AMPARs are glutamate-gated ion channels composed of combinations of the tetrameric subunits GluA1–4. AMPARs mediate much of the rapid, excitatory neurotransmission in the brain and are integral to LTP^74^. The composition of subunits has important consequences for the role of AMPAR in synaptic plasticity and typically AMPAR antibodies target GluA1 and GluA2 subunits^75^. AMPAR antibodies cause reductions in AMPAR expression with changes to their synaptic localisation through receptor internalisation and degradation^69,76^. Reductions in AMPAR-mediated currents are seen with alterations in the patterns of action potential firing and an increase in intrinsic excitability of neurons likely due to a compensatory decrease in inhibitory synaptic transmission^75,76^. Haselmann et al. demonstrated antibody-mediated internalisation of GluA2-containing AMPARs with compensatory insertion of mostly GluA1-containing AMPARs^77^. The subsequent LTP impairments were hypothesised to be secondary to the reduced availability of extrasynaptic AMPAR, on which LTP is dependent^77,78^. Alongside the LTP changes, they found in vivo impairments to learning and memory; this was the first animal model to recapitulate the severe memory impairments typical of AMPAR encephalitis^77^.

Although ubiquitous, GluA1/2 and GluA2/3 are particularly expressed in the hippocampal and limbic regions, and as such these regions are particular targets for AMPAR antibodies^69^. Indeed, in the vast majority brain MRI is abnormal in the acute phase, often showing bilateral temporal lobe enhancement, reflecting areas of greatest AMPAR density^71^. Given AMPARs are found throughout the brain, albeit at lower concentrations than in the limbic regions, autoantibody binding in other regions could account for the marked heterogeneity seen in clinical profile, with the more generalised distribution also underpinning the global atrophy and hypometabolism reported in some cases of AMPAR encephalitis^79^.

## GABA_A_R encephalitis

GABA_A_R antibody-mediated encephalitis has a broad clinical phenotype affecting all ages of both sexes^80,81^. The largest case series to date confirmed seizures as the most frequent symptom with altered cognition evident in two-thirds of patients^81^. While the clinical phenotype has yet to be fully characterised, greater variability in presentation is evident with memory deficits not ubiquitous and cases without seizures also described^82,83^. It is possible that the variation in clinical presentation of GABA_A_R antibody encephalitis across these series may be due to the differences in subunit specificity of cell-based assays used to detect the antibodies. Studies using the α1 and β3 subunits^80,81,84^ have tended to find a more restricted phenotype than those using the α1, β2 and γ2 subunits^82^, although differences may also reflect the different nature of the patient populations whose samples were tested in these studies.

### Mechanisms underlying cognitive impairment

GABA receptors are the major mediator of inhibitory synaptic transmission in the CNS. GABA_A_ receptors are ligandgated chloride ion channels, underpinning fast synaptic inhibition, while GABA_B_ receptors are G-protein coupled receptors modulating slower inhibitory transmission. Autoantibodies to GABA_A_Rs are generally IgG1 and those which target the extracellular epitope of the γ2, α1 and β3 subunit cause reduced synaptic and extrasynaptic GABA_A_R with consequent reductions in inhibitory postsynaptic currents in vitro^80,85^. Mutations to the GABA_A_R reducing expression levels cause generalised epilepsies but there are, as yet, no animal studies which demonstrate the impact of GABA_A_R antibodies in vivo^86^. However, in the acute phase brain MR imaging is commonly abnormal; 77% show multifocal, asynchronous grey and white matter changes most often in the temporal and frontal lobes^81^. These widespread changes reflect the extensive distribution of GABA_A_R which, along with the likely presence of additional anti-neuronal antibodies, may underpin the heterogeneity in presentation of GABA_A_R encephalitis^87^.

### GABA_B_R encephalitis

GABA_B_R encephalitis was first described in a case series of 15 patients characterised by seizures and memory deficits^88^. Older adults are most affected and there is a strong association with small cell lung cancer, occurring in up to 50% of patients and associated with poorer outcomes^89^. While more recent clinical descriptions have expanded the clinical phenotype, cognitive impairment and seizures remain the central symptoms, almost universally affecting patients in the acute phase but the nature of neuropsychological impairments have not been examined in detail^90^. Interestingly, this recent case series identified a subset of patients with GABA_B_R encephalitis presenting with a “rapidly progressive dementia” with subacute cognitive impairment in the absence of seizures^90^. Prognosis is often poor, with a median survival of 17 months, and the long term outcomes for GABA_B_R encephalitis have yet to be studied^90^.

### Mechanisms underlying cognitive impairment

Antibodies associated with GABA_B_R encephalitis are predominantly of the IgG1 subclass targeting the extracellular domain of the B1 subunit^88^. Autoantibodies to the GABA_B_R act to inhibit channel function rather than internalise or deplete cell surface receptor levels^91^. In line with the clinical phenotype, knockout GABAB1R mice exhibit spontaneous seizures with marked memory impairment^92^. The GABA_B_ receptor is mainly expressed in hippocampus, amygdala, thalamus, and cerebellum reflecting the common MTL abnormalities seen in imaging during the acute phase of encephalitis^93^.

### Beyond autoimmune encephalitis

While the impact of neuronal autoantibodies on cognition is well established within each encephalitic syndrome, their role outside this context is less clear. However, there is accumulating evidence to indicate these antibodies (particularly NMDAR antibodies) may be of relevance outside clinically defined encephalitis, in patients without evidence of frank encephalopathy. All NMDAR antibodies, irrespective of immunoglobulin class and donor source, demonstrate pathological potential; in vitro (and, to a lesser extent, in vivo) instigating NMDAR internalisation and dysfunctional glutamatergic signalling^94^. However, this is not equivalent to asserting that all such antibodies are potentially encephalitogenic and this distinction must be held in mind^95^.

Serum NMDAR antibodies, of uncertain clinical relevance, are found in appreciable numbers in healthy controls, which is a challenge to the view that they are universally pathological. In one such study serum NMDAR antibodies of all isotypes occurred at an overall frequency of about 10%, increasing with age but not differing according to disease status; NMDAR IgG remained relatively rare however, detectable in around 1%. Similar results were reported for antibodies to other antigens, although these were much less common than NMDAR antibodies^96^. Notably, different assays also appear to have different sensitivities for detection of neuronal autoantibodies. Live cell-based assays, in which sera or CSF is applied to recombinant HEK cells expressing the antigen of interest before fixation, appear to detect many more positive specimens than do fixed assays, in which the sera or CSF is applied after fixation. Nonetheless, while live CBAs do detect antibodies that demonstrably bind their target, the clinical relevance of the results is less clear—that is, the increased analytical sensitivity of these assays may come at the expense of clinical specificity^97,98^.

In part because of the issue of non-specificity of serum autoantibodies to neuronal antigens, diagnostic criteria for autoimmune CNS disorders place much emphasis on paraclinical investigations which are required to determine the clinical relevance of a positive antibody, such as MRI or EEG. Neither in consensus criteria for autoimmune encephalitis^99^ or for autoimmune psychosis^100^ can a positive serum antibody on its own lead to a diagnosis of probable antibody-mediated disorder.

It has been suggested that some aspect of blood-brain barrier permeability is one factor which can determine the clinical relevance of a positive serum antibody, with numerous studies showing autoantibody-mediated neuropsychiatric symptoms to be dependent on blood-brain barrier permeability^101–103^. Indeed, in SLE rodent models, infusion of NMDAR antibodies (targeting the NR2 rather than NR1 subunit) only caused cognitive impairment where the blood-brain barrier was disrupted^104^. It also remains possible that impaired blood-brain barrier integrity, or other mechanisms, could allow formation of antibodies in patients with neuronal decline via recognition of these neuronal antigens for the first time^105^. We review the evidence for a role of autoantibody-associated cognitive impairment in various common disorders.

### Cancer

Paraneoplastic neurological syndromes are immune-mediated disorders triggered by tumours driving the immunization process. Many of the autoimmune encephalitis-associated antigens are expressed by tumours, and paraneoplastic neurological syndromes can be associated with neuronal autoantibodies targeting these tumour-expressed neuronal antigens (such as NMDAR antibodies with teratomas and CASPR2 antibodies with thymomas). However, the association between tumours, neuronal antibodies and cognition has recently been explored beyond the context of limbic encephalitis. In a retrospective study, neuronal antibodies were observed in almost a quarter of cancer patients tested and cognitive deficits were found to be significantly more common in those with a positive serum neuronal antibody^106^. The antibodies most commonly identified were IgA or IgM NMDAR antibody, and the level of cognitive impairment was related to the degree of blood-CSF barrier disruption as indexed by the cerebrospinal fluid/serum albumin quotient^106^. These findings were then replicated and extended in a prospective study of melanoma patients^107^. Importantly, all patients underwent a comprehensive cognitive assessment that was performed blinded to antibody status. Melanoma patients with neuronal autoantibodies (mostly serum IgA and IgM NMDAR antibodies) showed more than threefold higher odds for cognitive impairment than melanoma patients without antibodies. Furthermore, the degree of cognitive impairment was correlated with the titre of NMDAR IgM or IgA antibody^107^. Affected cognitive domains included memory, attention and executive function indicating neuronal autoantibodies may have a role as both a pathophysiological factor and a potential biomarker for cognitive impairment^107^. However, future studies are needed to determine whether the observed cognitive impairments in antibody-positive patients are specific to cancer and whether antibodies themselves are pathogenic or rather indicate pathophysiological states leading to cognitive decline.

### Viral encephalitis

It is now established that neuronal autoimmunity—principally to NMDAR but other antigens have also been implicated—can be initiated by herpes simplex encephalitis (HSE)^108,109^. In up to 90% of so-called ‘relapses’ of HSE, where the clinical picture is frequently dominated by cognitive dysfunction, the aetiology is now understood to represent a “secondary autoimmune encephalitis” responsive to immunotherapy; indeed autoimmune encephalitis is thought to occur in around a third of HSE patients^58^. One obvious factor potentially responsible for initiation of autoimmunity is the gross neuronal destruction and subsequent epitope exposure caused by HSV infection. Indeed, other CNS viral infections are also known to initiate neuronal autoantibody production^109^. However, history of non-encephalitic HSV infection is also more common in NMDAR encephalitis^110^ suggesting molecular mimicry may also play a role^111^. Interestingly in one study, even in patients who did not develop frank encephalopathy after HSV infection, CSF NMDAR antibodies were interpreted to be predictive of the degree of improvement in cognitive function in the recovery phase of HSE^112^. However, in this study, there was no difference in cognitive performance between NMDAR antibody positive and negative patients at any time during follow-up; rather, there was only a significantly greater improvement of cognitive scores in the antibody negative group, driven by recovery from a worse baseline performance for these patients compared to that of the NMDAR antibody positive patients. Moreover, the relatively impaired performance of NMDAR antibody negative patients at baseline was driven by four outliers with particularly impaired performance^112^. Given the significant impact of post-HSE cognitive impairment on functioning and quality of life, and the lack of clarity of these results, attempts at clarifying the possible prognostic significance of NMDAR antibodies in this patient group are of continued interest. Overall it appears that secondary neuronal autoimmunity following other kinds of brain tissue damage could have a role in shaping the extent of cognitive dysfunction following an acute event. One such obvious example of brain tissue damage, amenable to study by virtue of its frequency, is stroke.

### Stroke

Serum neuronal antibodies are detected in up to one-fifth of patients following acute stroke^113^. However, there is as yet no consensus regarding the relevance of these antibodies in this clinical population. One recent study did not find any association of serum neuronal antibody seropositivity with functional outcome or clinical features in acute stroke^114^. Another large study did not find an association with seroprevalence per se, unless the group was stratified by NMDAR antibody titre, when high antibody titre was found to correlate with poor functional outcomes^115^. In addition, NMDAR antibody seropositive patients had an increased risk of secondary vascular events or death. The integrity of the blood-brain barrier may also be relevant in stroke: in patients with acute ischaemic stroke, NMDAR antibodies were associated with larger stroke lesions in patients with a “leaky” blood-brain barrier, as indicated by APOE4 status, and conversely, in patients with an intact blood-brain barrier NMDAR antibodies were associated with smaller stroke lesion size^113^. Thus, the relationship between autoantibodies and outcomes following stroke is not linear but serum NMDAR antibodies may be of particular relevance where they are found at high titre and/or with a compromised blood-brain barrier. Although functional outcomes are in part driven by cognitive status following stroke, to date no study has investigated an association between antibody seropositivity and quantitative cognitive outcomes following stroke. This would be methodologically challenging due to the inherent variability in cognitive outcomes with lesion heterogeneity but could offer the potential for new insights into the impact of neuronal antibodies outside encephalitis.

### Psychiatric disorders

Psychiatric symptoms are hallmarks of many of the autoimmune encephalitic syndromes, in some cases occurring in the absence of the other clinical symptoms^116^. This has caused considerable interest in investigating a potential role for neuronal antibodies in the pathogenesis of psychiatric syndromes. Various case-control studies have produced conflicting results, but a systematic review and meta-analysis found that serum NMDAR antibodies were three times as common in patients with schizophrenia, schizoaffective disorder, bipolar affective disorder or major depressive disorder compared to controls^117,118^. This finding was supported by a more recent large case-control study indicating that serum NMDAR antibodies were more prevalent in patients with first episode psychosis than in the healthy controls, although this was not the case for the other antibodies tested; LGI1, GABA_A_R and VGKC-complex^119^. However, in the psychiatric patient population, the clinical characteristics between antibody positive and antibody negative patients appear similar and to date the clinical significance of this increased prevalence is unclear^120^.

Cognitive impairment is a central feature of schizophrenia with the dysfunction extending across domains of memory, attention and executive function^121^. These deficits occur before the onset of psychosis and remain stable throughout the course of the disease^122,123^. Glutamate receptor hypofunction has been hypothesised to underlie this cognitive impairment and more recently NMDAR autoimmunity has been implicated. Indeed, a recent study found that first episode psychosis patients with schizophrenia who had a positive serum NMDAR antibody exhibited greater cognitive impairments in all domains relative to controls^124^. Furthermore, serum antibody level was inversely correlated with scores in verbal and learning memory, working memory and speed of processing. In this clinical population, the pathogenicity of NMDAR antibodies also appears to relate to blood-brain barrier integrity^101,125^. Increased blood-brain barrier permeability is known to associate with Toxoplasma gondii exposure in human cohorts and NMDAR antibody seropositivity (to the NR2 subunit) in schizophrenia was associated with higher degrees of cognitive impairment where it coexisted with Toxoplasma gondii exposure^125^. While these findings are of great interest, no firm conclusions can be made without replication on a larger scale.

### Dementia

Neuronal autoantibodies have also been detected at relatively high frequencies in a number of dementia syndromes; in one study increased prevalence of NMDAR antibodies, predominantly IgA and IgM, was demonstrated across all types of dementia (16% vs 2% in controls)^126^. While it remains unclear whether these autoantibodies have a primary pathogenic role or reflect a response to neuronal damage, there is some evidence to support a possible role in mediating cognitive symptoms. Patients with neurodegenerative disease such as Parkinson’s disease have been found to have serum NMDAR antibody frequencies in the range of controls unless there is evidence of dementia e.g., dementia with Lewy bodies or Parkinson’s disease dementia^126^. Although no such association was found in a more recent study of Parkinson’s disease with dementia, given the cognitive impairment in the studied group was modest (mean MMSE 25) this merits replication^127^.

The prevalence of serum NMDAR antibodies is not uniformly distributed across dementia subtypes. Disproportionately high frequencies of positive antibodies (>60%) are found in ‘unclassified’ or ‘atypical’ dementias^102,126^. These patients frequently had subacute onset with rapid progression or fluctuation and an inflammatory CSF, often showing reversibility when treated with immunotherapy^126,128^. In a recent meta-analysis, we reported that both IgG and IgA/M serum NMDAR antibodies were more prevalent in atypical dementias vs healthy controls, while there was no difference for all-cause dementia. However, the total number of studies was small and “atypicality” was inconsistently defined and in some studies may have been done so post-hoc, necessitating caution in interpretation of this intriguing result^129^.

The term “autoimmune dementia” has been proposed to describe this subacute cognitive impairment responsive to immunotherapy and, while its prevalence is unclear, there is growing suspicion that many cases may go undiagnosed, overlooked as primary neurodegenerative dementias^130^. In a study of 56 patients, a third of those who responded to immunotherapy, with notable improvements in all cognitive domains, had been initially diagnosed with a neurodegenerative or prion disorder^131^. Numerous case reports have illustrated the potential for antibodies to produce a phenocopy of established dementia syndromes, with misdiagnoses seen in cases of both NMDAR and LGI1 encephalitis mimicking atypical neurodegenerative dementias^11,37^. Given the reversible nature of autoimmune dementia, potential misdiagnoses of this nature could be catastrophic and all efforts to avoid them must be made.

### Neurodevelopmental implications

Placental transfer of IgG antibodies during gestation is a well-established phenomenon, with these antibodies also having the potential to penetrate the foetal blood-brain barrier during specific developmental windows^132,133^. It is therefore perhaps unsurprising that *in utero* autoantibody exposure has been implicated neurodevelopmentally as a pathogenic factor altering the cognitive development of the foetus. CASPR2 is known to have a critical role in neurodevelopment, is highly expressed in the proliferating zones and is necessary for dendritic spine development and arborisation, integral to neural circuit assembly^134^. In *CNTNAP2* knockout mice, which lack the predominant CASPR2 isoform, there are abnormalities in neuronal migration and reduced inhibitory GABAergic neurons causing a typical autistic phenotype with spontaneous seizures^135^. This phenotype is mirrored in paediatric patients with homozygous *CNTNAP2* mutations who lack CASPR2^136^. Mice exposed to CASPR2 antibody in utero show abnormal cortical migration and development with reduced glutamatergic synapses, increased microglial activation and decreased hippocampal inhibitory neurons^137,138^. The offspring showed subsequent long-term behavioural sequelae with repetitive behaviour and impairments in sociability and flexible learning^137,138^. Prevalence of CASPR2 antibodies was markedly higher in a subgroup of mothers with autistic children (37%) than in the control groups (8–12%)^137^. These results were not replicated in a Danish cohort study but CASPR2 antibodies were found more frequently in the mothers of children with “mental retardation or disorders of psychological development”^139^. This study did not find a significant association between maternal NMDAR antibodies and child cognitive development but a murine model has demonstrated reduced density of NMDAR in neonates of mothers with NMDAR antibodies with associated neuropathological changes and greater postnatal mortality and chronic increased hyperactivity^140^. An association between maternal lupus, with anti-dsDNA antibodies cross-reacting with the NR2A/NR2B subunits of the NMDAR, and neurocognitive problems in the offspring has been reported, with the offspring showing deficits in behaviour, memory and learning^141^. dsDNA-specific NMDAR antibodies injected into pregnant dams caused thinned, disorganised cortex in offspring with subsequent cognitive impairments^142^. While there is no evidence of overlap with the NR1 NMDAR antibodies seen in encephalitis it illustrates the potential for these antibodies to also exert an effect on neurodevelopment^143^.

### Conclusions and future directions

Each neuronal antibody exerts a distinct mechanistic effect and while the downstream effects all include cognitive dysfunction, the affected domains vary between subtype. However, there is much work to be done in fully characterising both the acute and chronic impairments of the encephalitic syndromes; current descriptions tend to be mostly qualitative and for the less common subtypes data is sparse.

Outside of encephalitis, pathogenicity of the neuronal antibodies may be contingent on factors including the integrity of the blood-brain barrier. Where this is compromised there is often evidence of secondary cognitive dysfunction; we postulate that blood-brain barrier disruption modulates much of the heterogeneity seen in the impact of neuronal antibodies outside of encephalitis. The site of autoantibody production may be equally important. In autoimmune encephalitis, it is likely that ongoing peripheral germinal centre reactions generate antigen-specific B cells. Subsequently, antigen-secreting cells that have differentiated from these B cells likely access the CNS, resulting in intrathecal production of pathogenic IgG^144^. It is not at all clear that the same process is occurring in the non-encephalitic situations described in this review. Indeed, blood-brain barrier disruption might be an important factor in some of the situations described above precisely because there is no intrathecal production of pathogenic antibodies. Furthermore, the description of unmutated yet functional (and potentially pathogenic, despite low affinity) NMDAR antibodies raises the possibility that, outside of the encephalitis context, the clinical relevance and/or pathogenicity of neuronal autoantibodies might in fact arise from the so-called ‘healthy’ naïve B cell repertoire^145^. Differences in epitope specificity, antibody titres and duration of interaction and initial immunising stimulus may also all have relevance in distinguishing encephalitis cases from non-encephalitis cases where the antibodies nonetheless may have some pathogenic role. An alternative, and underexplored, perspective is that neuronal autoantibodies (particularly “natural” antibodies with different binding properties) could have an adaptive physiological role; recent animal (and to some extent human) work suggests that NMDAR antibodies could be produced in response to stress as a mechanism to reduce anxiety/depressive behaviours, possibly via NMDAR antagonism^146^. It is conceivable that there is an analogous role in preventing, for example, excitotoxicity-mediated neuronal damage and cognitive impairment in some circumstances, although this remains to be explored.

Crucially, there is very little evidence currently for or against the possibility that immunotherapy could be an effective treatment in any of these non-encephalitis situations; the main notable exceptions are in cases of atypical or “autoimmune dementia”—and in some of these cases the demarcation from autoimmune encephalitis is far from clear^126,128,131^. The possibility that specific immunotherapies could have a role in treating cancer-associated cognitive impairment, or as a treatment to prevent the progression of cognitive impairment in neuronal antibody-positive post-HSV encephalitis patients, for example, warrants further evaluation.

Non-IgG NMDAR antibodies, in particular, have been implicated in the cognitive impairment seen in a multitude of disorders including cancer, dementia and schizophrenia. While NMDAR antibodies have been shown to universally have pathogenic potential^94^, the clinical consequence may vary with isotype. It is well established that the IgG isotype can cause NMDAR encephalitis while IgA NMDAR antibodies have been implicated in a more insidious cognitive impairment^128^. Indeed, the frequencies of NMDAR antibodies detected in dementia, cancer and stroke are more than twofold greater for IgA or IgM isotypes than IgG^102,106,113^. We suggest that these isotypes may be relevant for understanding cognitive impairment outside of encephalitis. While the potential exists for all the neuronal antibodies described to impact cognition, further work is needed to characterise this. In the future this could pave the way for novel, immunologically-based therapeutic options to treat cognitive impairment, with potentially transformative implications.
